# 

**Published:** 1887-02-12

**Authors:** 


The Hospital.

An Institution, Family, and Parochial Journal of Hospitals, Asylums, and all Agencies for the Care of the Sick, Criticism and News.

SATURDAY, FEBRUARY 12TH, 1887.

### The Literary Prize.

A PRIZE of **Two Guineas** will be given every month for the best story or incident based upon hospital, or asylum, or student life, or any incident connected with the professions of medicine or nursing.

### The Quilter Prizes.

### HOSPITAL HOUSEKEEPING AND MANAGEMENT.

We offer two prizes of Ten Guineas and Five Guineas respectively for the two best reports on the Expenditure of any single hospital, such reports to give all the details concerning the cost of food and stimulants, brought out in detail in the table on page ii of the Special Supplement we published last week, together with a critical examination of the various recommendations contained in the report of the Special Committee of the Newcastle Infirmary, and the addition of the figures to the following heads of expenditure. Hospital secretaries may procure a printed form of return, containing all the particulars to be filled up by competitors, on application to the Editor.

1. *Domestic Expenses.*—Soap and Soda; Laundry, Repairs, and Washing; Water Rate; Coals, Coke, and Wood; Gas and Oil; Bedding and Linen; Furniture, Earthenware, and Hardware; Uniform and Livery, for (*a*) Porters, (*b*) Sisters and Nurses.

2. *Surgery and Dispensary.*—Drugs; Surgical Dressings and Bandages; Instruments, Artificial Limbs, and Trusses; Mineral Waters; Dispensary Sundries.

3. *Salaries and Wages.*—(*a*) Management, including Secretary Superintendent, Clerks, and Collectors (including commission); (*b*) Nursing Department, Matron or Lady Superintendent, Superintendents of Nursing, Sisters, Nurses, Extra Nurses, Ward Maids and Scourers; (*c*) Maintenance of Porters and Servants (not included in *a* and *b*).

4. *Management Expenses.*—Printing and Stationery; Advertisements; Postage; Special Appeal for Funds; Annual Court and Festival Dinner; Auditor's Fee; Banker's Interest.

5. *Rates and Insurance* (exclusive of Water and Gas rates).

6. *Pensions*, showing to which Department they belong.

7. *Repairs and alterations of Structure.*—(*a*) General, including flooring and painting; (*b*) Extraordinary, including building.

8. *Miscellaneous Expenses* (*i.e.*, all other items to be given in detail).

All the figures are to be those for the year 1886, and the totals must agree with the figures published in the annual report, and copy of which must be sent in with the manuscript. Competitors must enclose their name and address, must write on one side of the paper only, and give the name of the hospital they have selected. No competition should occupy more space than that represented by three pages of printed matter in The Hospital.

All competitions must be sent in not later than the 31st of March next, addressed to the Editor, The Hospital, Norfolk House, Norfolk Street, London, W.C., and it is hoped that particulars may in this way be supplied from every British Hospital.

### THE CHILDREN'S CORNER.

### OUR PASTIMES.

**Children's Quarterly Prize Competition.**
Began 1st Jan., 1887 ; ends 26th Mar., 1887.

PRIZES.
FOR MOST CORRECT ANSWERS.

| | | | |
|---|---|---|---|
| 1st .. | .. Thirty Shillings | 3rd .. | .. Ten Shillings |
| 2nd .. | .. Twenty " | 4th .. | .. Five " |

FOR NEATEST ANSWERS.
Five Shillings.
FOR BEST LITTLE LETTERS.
Ten Shillings.

**Seventh Competition.**

No. 37. FIGURE-WORDS.
Two thousand one hundred, each divided by one,
Names a person who causes both anger and fun.

No. 38. NUMERICAL ENIGMA. I am one of twelve children, and eight letters will spell my name. My 8,2,6,4 is my parent; my 3,4,6,8 is what donkeys do ; my 1,5,7 is what muffs, boas, etc., are made of ; my 1,2,6,4 means terror ; my 1,5,7,8 means rage ; my 7,6,8 applied to light, means beam.

No. 39. CONUNDRUM. When one nigger dies, what do the others do ?

* * * * No. 40. SQUARE WORD. A vessel that goes to sea ;
* * * * to engage for wages ; the most useful of metals ;
* * * * what we write with.
* * * *

No. 41. GEOGRAPHICAL PUZZLE.
In ten not in four,
In much not in more,
In Ann not in Moll,
In Mary not in Poll,
In none not in all,
In Saul not in Paul.
My whole is a well-known river.

No. 42. CONUNDRUM. A gentleman was looking at a portrait and said, "Brothers and sisters have I none, but this man's father is my father's son." Whose portrait was he looking at ?

**Results of Fifth Competition.**

No. 28. CONUNDRUM. What is the riddle of riddles ? Here the guessing faculties of my little friends were taxed. Alsie fancies it must be the well-known Sphinx's Riddle ; Bismarck offers as the solution, "bad penmanship," because, he says, you "can't make it out" ; while Isobel suggests "truth." Has little Isobel been reading Bacon's *Essays*, and remembered the opening words of one, "What is truth ? asked jesting Pilate." Life, however, is the riddle of riddles, because everybody must *give it up*.

No. 29. CHARADE. The answer here is Love-letter.

No. 30. BURIED PROVERB. This is "Birds of a feather flock together."

No. 31. CONUNDRUM. The vessel used by a chemist most resembling an answer, is, of course, a *retort*.

No. 32. LATIN PUZZLE. The "translation" of this is "Dick for his supper has veal and ham."

*All right*—Tim, Scrooge, A. C. K.; *Four right*—Alsie, Peeler, Mikado, P. S., Dot, Joe, Paul Methven ; *Three right*—Skye, Prospect, Isobel, Ellie, Little Mary, Bismarck, Ariadne, Florrie.

**Letters from Little Folk.**

So many nice letters have come to hand this week, that it is a matter of difficulty to make a selection for the printer. Here is one from "Mikado," who has been paying a visit to a hospital ; see how kind and thoughtful she has been :—

DEAR MR. EDITOR,—I went to a Hospital to see the Children's Ward, and to take them a scrap-book which I had made. I gave it to a little boy, as the nurse said he would have to be in a long time. They all looked so pale and quiet : I wished I had got something for each of them. They had got some very pretty pictures on the walls, and each little child had on a warm red flannel jacket. I think it must be very nice to be a nurse in a children's ward, to try and amuse those that are better. I am going to save some of my pocket-money to buy some toys to take them next time I go. I think the little boy would like some soldiers or a Noah's ark, when he gets tired of the scrap-book. Yours truly,

12, Upper Tichborne Street, Leicester. "MIKADO."

My young friend Halford was, I am sure, very lucky to be the recipient of such beautiful presents on his ninth birthday. Here is what he tells me about them :—

DEAR MR. EDITOR,—I said that I would tell you about the presents that I had on my birthday. Father gave me a fine set of tools, and mother a box of soldiers, and a cannon that shoots peas. And I had a Prayer Book and a scarf, and a very amusing book called *Tippoo*, which I like very much. I want to know if you would mind my only putting H. instead of H. G. B. Yours truly, H.

The Lodge, Porchester Square, W.

Here is a note from Tim, who has been to the play :—

DEAR MR. EDITOR,—I think I must tell you about *Alice in Wonderland*, which we saw the other day. We have read *Alice in Wonderland* and *Alice through the Looking-glass*, and it made it more interesting because we could understand what the different people said. The animals we liked best were the Gryphon, the Cheshire Cat, and the Dormouse, while Alice was exactly like the little girl in the picture.

Your little friend, TIM.

25, Dornton Road, Balham.

**Letter Prize.**

One mark each: Isobel, Skye, Peeler, Tim, H., Mikado, Ellie, Florrie, and Scrooge.

**Notices to Correspondents.**

ISOBEL.—Thank you kindly for the puzzle.

SKYE.—I am so pleased to hear you attend an Art school. Drawing and painting are delightful accomplishments ; they elevate our tastes, and enrich our minds with a love of all that is truly beautiful. Thank you for enclosures.

MIKADO.—Many thanks for your welcome contributions.

P. S.—I am much obliged for the conundrum.

ELLIE.—Thank you for the puzzles. Your notes are always so nicely written.

FLORRIE.—I read your letter about school with great interest. I am sure you will like Latin when you get to understand it a little better. Thank you kindly for the puzzles.

SCROOGE.—I shall be delighted to hear about the concert.

Answers, having the actual name and address (to which a pseudonym may be added for publication), must have "The Children's" Coupon attached (see advertisement pages), and be addressed to the "PUZZLE EDITOR," THE HOSPITAL, Norfolk House, Norfolk Street, Strand, W.C., not later than Thursday next.

### AMUSEMENTS WITH PROFIT.

### PRIZE COMPETITIONS.

**Quarterly Prize Competition.**
Began 22nd January ; Ends 16th April.

**A Prize of Three Guineas**
will be awarded to the Competitor who shall have sent in the largest number of correct or best answers. No one will be permitted to win the Three Guinea Prize twice in any one year, but we shall award annually an additional

**Prize of Five Guineas**
to the competitor who shall have sent in the largest number of correct or best answers during the twelve months.

**No. 7. Double Acrostic.**

A *teacher* my *primals* once implied,
Now mostly a medical term ;
My *finals* denote a *lady*, tried,
A hospital chief, controlling, firm.
An English seat of learning in the *North* you'll find ;
One of the towns by which *South Russia's* coast is lined ;
In Finland's icy *gulf* this mighty fort is found ;
Old *Rome* stood on my banks and made my name renown'd ;
Mid the *Canadian* lakes my name you'll surely trace ;
An old *French town*—Jean d'Arc died in its market-place.

**No. 8. Buried Composers.**

My brother, who was shot by a pagan in India, was once in the Punjaub erecting bridges. He bought a howdah and elephant, which cost a good deal, from a cast-off lot owned by a nabob. One day, he went out to stab a cheetah ; I never did see such a large one. We were far nearer than was pleasant, when I saw its tail wag nervously, and I said, with a gasp, "Oh, run for your life ere we be rushed at," when my dog luckily killed it. (Credit will be given only for answers which reveal the buried names of *all* the composers to be found in the above.)

**Answers.**

No. 3. PLAY ON WORDS.

The translation here is, "Theodore Hook was one day walking with a friend. 'Behold,' exclaimed the friend, 'a man whom I know, and who calls himself a teetotaller, overcome by drink.' 'Very likely,' replied Hook, 'for you know that he with tea may be tipsy, (*i.e.* "ipse," with "te," may be "tipse" = "te-ipse.")

Correct answers have been received from A. M. E., Condorcet, Ostrich, Ellice.

No. 4. DOCTOR.

Some of our competitors have taken "definition" in a wider sense than we intended. Several of the answers, however, were tersely and cleverly pointed.

Best answers have been received from Patient, H., Paddy, K. M., Condorcet, H. J. C., Gretta, Ostrich, Mater, A. M. E., and Perseverance, Ellice.

Answers must be addressed to THE PRIZE EDITOR, AMUSEMENTS WITH PROFIT, THE HOSPITAL, Norfolk House, Norfolk Street, Strand, W.C., not later than Saturday next. The full name and address must in all cases be given, but a *nom de plume* may be added for publication. Only one side of the paper should be written on. The "Amusements with Profit" Coupon (see advertisement pages) must be enclosed with replies.

### The In- and Out-Patients' Hospital Diary.

This Diary is so arranged as to make some portion of it appear every week, and the whole in each monthly part. It has been very difficult to compile, and corrections will at all times be gratefully received. It has been suggested that similar information about the larger Provincial and Scotch Hospitals would be useful to many readers. We should be glad to hear if this is felt to be the case.

### I.—GENERAL HOSPITALS.

| Hospitals and Terms of Admission. | Physicians, Surgeons, and Medical Officers, with Days and Hours of Attendance. |
|---|---|
| Charing Cross Hospital, West Strand, W.C. Sec., Mr. A. E. Reade<br>Urgent cases are immediately admitted as in-patients. Subscriber's letter expected in other cases<br>Out-patients admitted without subscriber's letter first time; for continued attendance they must be obtained from the Secretary | *In-Physicians*, Drs. Pollock, M. Th.; Green, W. S.; Bruce, Tu. F. Hour, 1.30<br>*In-Surgeons*, Messrs. Barwell, M. Th.; Bellamy, Tu. F.; Bloxam, W. S. Hour, 2<br>*Out-Physicians*, Drs. Wilcocks, M.Th.; Abercrombie, Tu. F.; Lubbock, W. S.; Murray, W. S. Hour, 1.30<br>*Out-Surgeons*, Messrs. Cantlie, M.Th.; J. H. Morgan, Tu. F.; Stanley Boyd, W. S. Hour, 1.30 |
| "Dreadnought" Hospital (Seamen's Hospital Society), Greenwich, S.E. Sec., Mr. W. T. Evans<br>Entirely free to seamen and to all accident cases | *In- and Out-Physicians*, Drs. Curnow, Tu. Th. S.; H. T. Griffiths, M.W.F. Daily, between 9 and 3<br>*In- and Out-Surgeons*, Messrs. W. J. Smith, G. R. Turner. Daily, between 9 and 3 |
| French Hospital, 10, Leicester Place, Leicester Sq., W.C. Sec., Mr. E. Rimmel<br>Free to urgent cases and to all foreigners speaking French | *In-Physician*, Dr. Vintras, Tu. S. 10<br>*In-Surgeons*, Sir W. MacCormac, Tu. 9; Mr. J. Késer, W. F. 10<br>*Out-Physician*, Dr. Vintras, Tu. S. 10<br>*Out-Surgeons*, Messrs. H. de Méric, M. Th. 10; J. Késer, W. F. 10 |
| German Hospital, 113, Dalston Lane, E. Sec., Mr. C. Feldmann<br>Free to natives of Germany, and others speaking German, and to all accidents and emergencies. Others by Governors' letters | *In-Physicians*, Drs. Weber, M. T. 2; Port, Tu. F. 9 a.m.<br>*In-Surgeons*, Drs. Lichtenberg and Burger, W. S. 2<br>*Out-Physicians*, Drs. Tuchmann, Tu. W. 2; Ludwig, M. Th. 2; Keller, Tu. W. F. 2; Philippi, M. Th. F. 2 (*Men*, Tu. Th.; *Women*, M. W. F.) |
| ——— Eastern Branch, 49, Mansell Street, Aldgate, E. | *Out-Physician*, Dr. C. Harrer, Tu. F. 8 to 10 a.m. |
| ——— Western Branch, 259, Oxford Street, W. | *Out-Physician*, Dr. M. Castaneda, M. T. 8 to 10 a.m. |
| Great Northern Central Hospital, Caledonian Road, N. Sec., Mr. W. T. Grant<br>Free to all, without letters of recommendation | *In-Physicians*, Drs. Cholmeley, Cook, and Burnett, M. Tu. W. Th. F. 2<br>*In-Surgeons*, Messrs. W. Adams, W. Spencer Watson, and J. Macready, M. Tu. W. Th. F. 2<br>*Out-Physicians*, Drs. Beale, M. Th. 2; Beevor, Tu. 2, S. 10<br>*Out-Surgeons*, Messrs. Lockwood, M. Th. 2; Makins, Tu. F. 2 |
| Guy's Hospital, St. Thomas's Street, S.E. Supt., Dr. C. J. Steele<br>Patients are admitted, if beds are available, without letters of recommendation, on application at the Hospital. Out-patients are seen free without letters of recommendation | *In-Physicians*, Drs. Pavy, Tu. F.; Pye-Smith, M. Th. S.; Taylor, M. Tu. Th. F. Hour, 1.30<br>*In-Surgeons*, Messrs. Bryant, M. Th. Durham, M. Th. F.; Howse, W. S.; D. Colley, M. Th. Hour, 1.30<br>*Out-Physicians*, Drs. Carrington, W.; Hale White, M.; Goodhart, M. Tu. Th. F. Hour, 12.30<br>*Out-Surgeons*, Messrs. Lucas, Th.; Golding Bird, S.; Jacobson, W.; Symonds, M. Hour, 12.30 |
| King's College Hospital, Portugal Street, W.C. Sec., Mr. T. Mosse Macdonald<br>Accidents and urgent cases free at all times. Other cases by Governors' letters, but where not obtainable, application may be made to the Secretary | *In-Physicians*, Drs. Beale, Duffin, Yeo, Tu. W. F. 1.30; Tu. W. F. S. 2<br>*In-Surgeons*, Mr. Wood, Sir J. Lister, Messrs. H. Smith, Cheyne, W. Rose, Barrow. Daily, 1.30<br>*Out-Physicians*, Drs. Ferrier, J. Curnow, N. I. C. Tirard; *Men*, Tu.Th.; S. 1; *Women*, M. W. F. 1<br>*Out-Surgeons*, Messrs. W. Rose, W. Cheyne; *Men*, Tu. Th. S. 1; *Women*, M. W. F. 1 |
| London Hospital, Whitechapel Road, E. Sec., Mr. A. Haggard<br>Accidents and urgent cases free; out-patients for dental and maternity departments need no recommendation; other cases by subscriber's order. Out-patients are required to attend only once, unless otherwise directed by the Physician or Surgeon | *In-Physicians*, Drs. Mackenzie, Tu. F.; Down, Tu. F.; H. Jackson, M. Th.; Sutton, M. Th.; Fenwick, Tu. F. 1.30<br>*In-Surgeons*, Messrs. Rivington, Tu. F.; Couper, Waren Tay, M. Th.; McCarthy, W.S.; Treves, Tu.F. Hour, 1.30<br>*Out-Physicians*, Drs. Sansom, Th.; M. Ralfe, M. Th.; Turner, Tu. F.; Warner, Tu. F.; Gilbart Smith, W. S. J. Anderson, W. S. Hour, 1.30<br>*Out-Surgeons*, Messrs. Mansell-Moullin, M. Th.; Eve, M. W.; Reeves, Tu. S.; H. Fenwick, Tu.F. Hour, 1.30 |
| London Temperance Hospital, Hampstead Road, N.W.<br>For treatment without alcohol. By letter or small payment | *In- and Out-Physicians*, Drs. J. Edmunds, M.; J. J. Ridge, Tu. F. Hour, 1.30<br>*In- and Out-Surgeons*, Mr. A. P. Gould, Th. 2.30 |
| Metropolitan Free Hospital, Kingsland Road, N. Sec., Mr. G. Croxton<br>Admission free to the sick poor, without any letter of recommendation. Urgent cases at all times | *In- and Out-Physicians*, Drs. Drysdale, Tu. F. 12; J. G. Dudley, W. S. 12; Fotherby, M. Th. 12; H. H. Tooth, Tu. F. 9; A. Haig, W. S. 9; A. Davies, M. Th. 9<br>*In- and Out-Surgeons*, Messrs. E. J. Chance, W. S. 9; Goodsall, Tu. F. 9; Walsham, M. Th. 9; A. A. Bowlby, F. 9; S. Paget, Th. 9; C. J. Thompson, daily, 9 |
| Middlesex Hospital, Berners Street, W. Sec. and Supt., Mr. A. O. D. Bartholeyns<br>Urgent cases admitted at all times; other cases by ticket. All medical cases must, in the first instance, be seen by the Resident Medical Officer, who attends at the hospital daily, at 11 a.m. | *In-Physicians*, Drs. Cayley, M. Th.; Coupland, Tu. F.; D. Powell, M.Th.; Finlay, Tu. F. Hour, 1.30<br>*In-Surgeons*, Messrs. Hulke, M. Th.; Lawson, M. Th.; Morris, Tu. F. Hour, 1.30<br>*Out-Physicians*, Drs. Fowler, Tu. F. 3.30; Biss, M. Th. 1.30; Pringle, W. S. 1.30<br>*Out-Surgeons*, Messrs. Andrew Clark, M. 1.15 (*Men*); Th. 1.15 (*Women*); Pearce Gould, F. 1.30 (*Men*); Tu. 1.30 (*Women*); J. B. Sutton, W. S. 9 |
| Miller Hospital and Kent Dispensary, Greenwich, S.E. Hon. Sec. Mr. W. Bristow. | *Physicians*, Drs. T. Creed, C. H. Hartt, G. Willes<br>*Surgeons*, Messrs. T. Moore, J. Poland, E. Clarke |
| North-West London Hospital, 18, Kentish Town Road, N.W. Sec., Mr. A. Craske<br>By subscribers' tickets or payment, but accidents and urgent cases free at all times | *In- and Out-Physicians*, Drs. W. C. Hood, J. Shaw, Edwarde H. Campbell. Daily, 1.30<br>*In- and Out-Surgeons*, Messrs. F. Durham, Collier, Jennings, J. Black. Daily, 1.30 |
| Ospedale Italiano, 41, Queen Square, Bloomsbury. Sec., Sig. L. Bonacina<br>Free to all, but Italians have preference | *In- and Out-Physicians*, Drs. M. Castaneda, Tu. F. 10 to 11; B. Sassella, M.Th. 10 to 11; Mr. J.W.B. Steggall, W.S. 4 to 5 |
| Poplar Hospital, 303, East India Dock Road, E. Sec., Lt.-Col. Feneran<br>Free to accidents and emergencies | *In-Surgeons*, Messrs. F. M. Corner, M. Brownfield, and Resident Surgeons<br>*Out-Surgeons*, Dr. J. D. Grant, W. S. Mr. T. E. Bowkett, Tu. F. 12 to 2 |
| Royal Free Hospital, Gray's Inn Road, W.C. Sec., Mr. J. S. Blyth<br>Admission free to the sick poor, without any letter of recommendation | *In- and Out-Physicians*, Drs. Cockle, M. Th.; Sainsbury, Tu. F.; West, W. S. Hour, 1<br>*In- and Out-Surgeons*, Messrs. Rose, M. Th.; W. Gant, Tu. F.; Barrow, W. S. Hour, 1 |
| St. Bartholomew's Hospital, Smithfield. Clerk, Mr. W. Cross<br>Admission free, without letter or ticket. Accidents and other urgent cases admitted at any time; ordinary cases of disease daily, between 9 and 10. Patients are seen for Electrical treatment on M. Tu. Th. and F. 1.30 | *In-Physicians*, Drs. Andrew, Church, Gee, Sir D. Duckworth. Daily, 1.30<br>*In-Surgeons*, Messrs. Savory, T. Smith, Willett, Langton, M. Baker. Daily, 1.30<br>*Out-Physicians*, Drs. Hensley, M. Th. 11; Brunton, Tu. F. 11; Legg, W. S. 11; N. Moore, Tu. W. Th. S. 9<br>*Out-Surgeons*, Messrs. Marsh, Tu. F.; 12; Butlin, M. Th. 12; Walsham, W. S. 12; Cripps and Bruce Clarke, daily, 9<br>*Casualty Physicians*, Drs. Davies, Tu. W. F. S.; O. Browne, M. Tu. Th. F.; Nias, M. W. Th. S. Hour, 9 |
| St. George's Hospital, Hyde Park Corner, S.W. Sec., Mr. C. L. Todd<br>Free to accidents and emergencies. Other in-cases by Governor's or subscriber's letter, but, in necessitous cases, this recommendation is not strictly insisted on. Letters are not required for out-patients. Vaccination on Th. at 11 | *In-Physicians*, Drs. Wadham, M. F.; Dickinson, M. Tu. Th. F.; Whipham, Tu.W. S.; Cavafy, Tu. S. Hour, 1<br>*In-Surgeons*, Messrs. Holmes, M. Th. F. 1, W. 1.30; Rouse, M. Th. F. 1, W. 1.30; Haward, Tu. Th. S. 1, W. 1.30<br>*Out-Physicians*, Drs. Ewart, M. F.; Owen, Tu. S. Hours, 10.30 to 12<br>*Out-Surgeons*, Messrs. Bennett, M. F.; Dent, Tu. S. Hours, 10.30 to 12 *Men*, F. S.; *Women*, M. Tu. |

### The In- and Out-Patients' Hospital Diary.

### GENERAL HOSPITALS.—*continued.*

| Hospitals and Terms of Admission. | Physicians, Surgeons, and Medical Officers, with Days and Hours of Attendance. |
|---|---|
| St. Mary's Hospital, Cambridge Place, Paddington, W. Sec., Mr. Pietro Michelli. In-patients are admitted by letter of recommendation. Urgent cases and accidents free at all times. Out-patients require a letter. Electrical treatment Tu. and F. 2 | *In-Physicians*, Sir Edward Sieveking, Tu. F. 1.45; Drs. Broadbent, M. Th. 1.45; Cheadle, W. 1.45, S. 9.30. *In-Surgeons*, Messrs. Norton, M. Th. 1.45; Owen, Tu. F. 1.45; H. Page, W. S. 1.45. *Out-Physicians*, Drs. D. B. Lees, W. S.; S. Phillips, M. Th; R. Macguire, Tu. F. Hour, 1.30. *Out-Surgeons*, Messrs. W. Pye, M. Th.; A. J. Pepper, Tu. F.; A. Q. Silcock, W. S. Hour, 1.30 |
| St. Thomas's Hospital, Westminster Bridge Road, S.E. Steward, Mr. F. Walker. Accidents and emergencies free at all times. Other cases by Governor's letter, but, where unobtainable, application should be made to the Steward by letter, or personally between 10 and 4. Vaccination on W. at 11.30 | *In-Physicians*, Drs. Bristowe, Tu. F.; Stone, M. Th.; Ord, M. Th.; J. Harley, Tu. F.; Gervis, Tu. F. Hour, 2. *In-Surgeons*, Messrs. Sydney Jones, Tu. F.; Croft, M. Th.; Sir W. MacCormac, M. Th. Hour, 2. *Out-Physicians*, Drs. Payne, Tu. F.; Sharkey, M. Th.; Gulliver, W. S. Hour, 1.30. *Out-Surgeons*, Messrs. Clutton, Tu. F.; Anderson, W. S.; Pitts, M. Tu. Hour, 1.30 |
| Tottenham Training Hospital, Tottenham, N. Director, Dr. Laseron. Free. Accidents and emergencies admitted at all times | *In-Physician*. Dr. Rasch, Tu. F. 3 to 5. *In-Surgeons*, Drs. Lichtenberg, M. Th. 3 to 5; E. H. May, M. Th. 3 to 5. *Out-Surgeon*, Dr. Lloyd Smith, M. W. 11 to 2; *Men* only, W. 7 to 8 p.m. |
| University College Hospital (or North London), Gower Street, W.C. Sec., Mr. N. H. Nixon. Accidents and emergencies free. All other cases by Governor's letter of recommendation | *In-Physicians*, Drs. W. Fox, Tu. Th. 2, S. 9.30; Ringer, M. W. F. 1.30; Bastian, M. 2.30, W. 10, F. 2.30; F. Roberts, Tu. 1.15, Th. 1.30, S. 9.30. *In-Surgeons*, Messrs. B. Hill, Tu. 2.15, W. 2, F. 2.15; C. Heath, M. W. F. 2; M. Beck, Tu. Th. S. 2. *Out-Physicians*, Drs. Gowers, W. S.; Poore, Tu. F.; Barlow, M. Th. Hour, 1.30. *Out-Surgeons*, Messrs. Barker, M. Th.; Godlee, Tu. F.; Horsley, W. S. Hour, 1.30 |
| West London Hospital, Hammersmith Road, W. Sec. and Supt., Mr. R. J. Gilbert. Accidents and emergencies free at all times. Other cases require a letter of recommendation. New cases must attend at 1.30 | *In-Physicians*, Drs. G. Rogers, D. W. Hood, F. Drewitt. *In-Surgeons*, Messrs. C. Keetley, F. Edwards, W. B. Clarke. *Out-Physicians*, Drs. Herringham, M. Th.; Ball, Tu. F.; S. Taylor, W. S. Hour, 2. *Out-Surgeons*, Messrs. Ballance, Tu. F.; Weiss, M. Th.; Wainewright, W. S. Hour, 2 |
| Westminster Hospital, Broad Sanctuary, S.W. Sec., Mr. S. M. Quennell. Accidents and urgent cases free at all times. Other cases require a letter of recommendation | *In-Physicians*, Drs. Sturges, M. Th. S.; Allchin, M. Tu. F.; Donkin, Tu. W. S. Hour, 1.30. *In-Surgeons*, Messrs. Cowell, M. Tu. Th.; Davy, M. Tu. F.; Macnamara, M. W. S. Hour, 1.30. *Out-Physicians*, Drs. De H. Hall, M. Th.; A. H. Bennett, Tu. F.; Murrell, W. S. Hour, 1.30. *Out-Surgeons*, Messrs. Cooke, M. Th.; Bond, Tu. F.; Barrow, W. S. Hour, 1.30 |

### II.—SPECIAL HOSPITALS.

### CANCER.

| Hospitals and Terms of Admission. | Physicians, Surgeons, and Medical Officers, with Days and Hours of Attendance. |
|---|---|
| Cancer Hospital, Brompton, S.W. Sec., Mr. W. H. Hughes. Entirely free to both in- and out-patients | *In- and Out-Surgeons*, Drs. A. Marsden, W.; H. Snow, M.; F. A. Purcell, F.; Messrs. F. B. Jessett, W.; C. Stonham, Th.; C. Jennings, Tu.; W. H. Elam, S. Hour, 2 |
| London Hospital, Whitechapel Road, E. | Daily, 1.30. *See* General Hospitals |
| Middlesex Hospital, Berners Street, W. Admission free | *In-Surgeons*, Messrs. Hulke, Lawson, and Morris. *Out-Surgeon*, Mr. Morris, Th. 1.30 |
| St. Saviour's Hospital, Osnaburg Street, N.W. Sec., Mr. E. Poitiers. By Governors' letters | *In- and Out-Physician*, Dr. A. Kennedy, 10.30 and 4 daily |

### CHILDREN'S DISEASES.

| Hospitals and Terms of Admission. | Physicians, Surgeons, and Medical Officers, with Days and Hours of Attendance. |
|---|---|
| Alexandra Hospital for Children, 18, Queen Sq., W.C. Sec., Mrs. Marsh. For hip disease | *Physician*, Dr. Steavenson. *Surgeons*, Messrs. H. Marsh Tu. F.; A. Bowlby, M. Th. Hour, 4.30. (No out-patients) |
| All Saints' Children's Hospital, 4, Margaret Street, W. For chronic joint diseases | *Surgeon*, Mr. N. Smith. (No out-patients) |
| Belgrave Hosp. for Children, 77 & 79, Gloucester Street, Pimlico. Hon. Secs., Capt. Stopford, and Rev. J. Storrs. By letters; accidents free | *In- and Out-Physicians*, Drs. W. Hope and W. Ewart, M. Th. 9. *In- and Out-Surgeons*, Messrs. C. T. Dent and Margerison, Tu. F. 9 |
| Charing Cross Hospital, West Strand, W.C. | *Out-Physician*, Dr. Nursby, W. S. 1.30 |
| Cheyne Hospital for Sick and Incurable Children, 46, Cheyne Walk, Chelsea, S.W. Sec., Miss D. Evans | *Physician*, Dr. G. V. Poore. *Surgeons*, Messrs. T. P. Bartlett and J. Macready attend alternate days in afternoons. (No out-patients) |
| East London Hospital for Children and Dispensary for Women, Glamis Road, Shadwell, E. Sec., Mr. Ashton Warner. By Governor's letter. Urgent cases and accidents are admitted free | *In-Physicians*, Drs. Donkin, M. Th.; Eustace Smith, T. F.; Crocker, F. *In-Surgeons*, Messrs. Calder, W. F.; Parker, W. F. *Out-Physicians*, Drs. Crocker, M.; Williams, Tu. Th.; Coutts, W. F. Hours, 1 and 3. *Out-Surgeon*, Mr. Dunn, Tu. F. 1, 3 |
| Evelina Hospital for Sick Children, Southwark Bridge Road, S.E. Sec., Mr. T. S. Chapman. Free, but Governor's letter takes precedence. In-patients (boys) between 2 and 10, (girls) between 2 and 12 | *In-Physicians*, Drs. F. Taylor and J. Goodhart. *In-Surgeons*, Messrs. H. G. Howse and R. C. Lucas. *Out-Physicians*, Drs. N. Tirard, M. Th. 9; F. Willcocks, Tu. F. 9. *Out-Surgeons*, Messrs. C. J. Symonds, S. 9; G. H. Makins, W. 9 |
| German Hospital, Dalston Lane, Dalston, E. | *In-Physician*, Dr. Rasch, M. Th. 9 |
| Great Northern Central Hospital, Caledonian Rd. N. | *Physicians*, Drs. Murray and Barnes, W. 2.30 |
| Grosvenor Hospital for Women and Children, Vincent Sq., S.W. Hon. Secs., Mr. A. J. Ram and Miss Day. By letter or payment | *In- and Out-Physicians*, Drs. R. Gibbons and Steavenson, Tu. W. Th. S. 2. *In- and Out-Surgeons*, Messrs. Smythe and Parker, Tu. W. Th. S. 2 |
| Hospital for Sick Children, 48 and 49, Great Ormond St., W.C. Sec., Mr. S. Whitford. By Governors' letters. If without letter, the case of the applicant is investigated. In-patients between 2 and 12; Out-patients under 12 | *In-Physicians*, Drs. W. Cheadle, Tu. F.; Sturges, W. S.; Barlow, M. Th. Hours, 9 to 11. *In-Surgeons*, Messrs. H. Marsh, W. S.; E. Owen, W. S. Hours, 9 to 11. *Out-Physicians*, Drs. R. J. Lee and Abercrombie, M. Th.; Lubbock and Money, Tu. F. Hours, 8.30 to 10. *Out-Surgeons*, Messrs. Morgan and Pitts, W. S. 8.30 to 10 |
| King's College Hospital, Portugal Street, W.C. | *In- and Out-Physician*, Dr. T. C. Hayes, W. F. 1 |
| Middlesex Hospital, Berners Street, W. Free. Children under 3 | *Out-Physician*, Dr. Duncan, W. S. 1.30 |
| North Eastern Hospital for Children, Hackney Road, E. Sec., Mr. A. Nixon. By free ticket or small payment | *In- and Out-Physicians*, Drs. W. Cayley, F. Turner, C. Semple, and W. Pasteur, M. W. Th. S. 1.30. *In- and Out-Surgeons*, Messrs. Waren Tay and Pollard, M. 1.30, F. 9 |
| Paddington Green Children's Hosp., Paddington Green. Sec., Mr. W. H. Pearce. Admission free; boys under 12; girls under 14 | *In- and Out-Physicians*, Drs. T. Lauder Brunton, Ogilvie, Tu. F.; G. Laycock, M. Th.; S. Phillips, W. S. Hour, 9.15. *In- and Out-Surgeons*, Messrs. W. W. Cheyne, M., S. Boyd, F. Hour, 9.15 |
| Royal Hospital for Children and Women, Waterloo Bridge Road, S.E. Sec., Mr. R. G. Kestin. Out-Patients pay 1*d.* on each attendance. Boys not admissible, as out-patients, over 12, or, as in-patients, over 8 | *In-Physicians*, Drs. Park, M. Th.; Gulliver, Tu. F.; Price, W. S. Hour, 2. *In-Surgeon*, Mr. W. H. A. Jacobson, M. Th. Hour, 2. *Out-Physicians*, Drs. Park, M. Th.; Dakin, W. F.; Hadden, Tu. S. Hour, 2. *Out-Surgeon*, Mr. E. O. Day, W. 2 |
| St. Mary's Hospital, Cambridge Place, Paddington | *Out-Physician*, Dr. M. Handfield Jones, M. Th. 1.30 |
| St. Thomas's Hospital, Westminster Bridge Road, S.E | *Out-Physician*, Dr. Cory, W. S. 1.30 |

